# Bell's palsy following COVID-19 vaccine administration in HIV+ patient

**DOI:** 10.1016/j.ajoc.2022.101259

**Published:** 2022-01-20

**Authors:** Caroline C. Mussatto, Jason Sokol, Neeti Alapati

**Affiliations:** a University of Kansas School of Medicine, 3901 Rainbow Blvd, Kansas City, KS 66160, USA; b Department of Ophthalmology, University of Kansas School of Medicine, 7400 State Line Rd, Prairie Village, KS, 66208, USA

**Keywords:** COVID-19 vaccination, Bell's palsy, Facial nerve palsy, Facial weakness, Vaccine reaction, Adverse event following immunization

## Abstract

**Purpose:**

COVID-19 immunizations are novel and there is widespread public concern for the lack of data on their potential adverse effects. Cases of Bell's palsy following COVID-19 vaccination were reported disproportionately in the vaccine group during phase 3 clinical trials and have now been reported multiple times post-licensure. The U.S. Food and Drug Administration has stated the frequency of Bell's palsy in the vaccine group is consistent with the expected background rate of Bell's palsy in the population but recommends “surveillance for cases of Bell's palsy with deployment of the vaccine into larger populations.“^1^ Here we present a case of Bell's palsy following Pfizer/BioNTech BNT162b2 COVID-19 vaccine administration in an HIV^+^ patient as a potential adverse event following immunization.

**Observations:**

A 60-year-old male with HIV presented to the emergency department for evaluation of left facial droop. He had received the first dose of Pfizer/BioNTech BNT162b2 vaccination approximately 42 hours prior to symptom onset. Physical examination in the ED revealed left-sided facial weakness with involvement of the forehead, inability to raise left eyebrow, and inability to close left eye with sensation and strength intact in bilateral upper and lower extremities. Physical examination in our outpatient ophthalmology clinic on day 2 following symptom onset was revealing for mild exposure keratopathy, 5 mm lagophthalmos and very poor Bell's reflex in the left eye with otherwise normal exam findings. These findings were judged to be consistent with uncomplicated Bell's palsy. He was provided ophthalmic lubricating ointment to use hourly, artificial tears as needed, moisture goggles and suggested to tape eyelids nightly in addition to standard systemic glucocorticoid and antiviral therapy. The patient's facial weakness and exposure keratopathy were completely resolved at approximately 90 days following symptom onset.

**Conclusions:**

Though there is insufficient evidence at this time to support any causal association between COVID-19 vaccines and Bell's palsy, the temporal relationship between vaccination and classic clinical features of Bell's palsy in our patient certainly raises suspicion for association with Pfizer/BioNTech BNT162b2 COVID-19 vaccination. It will be important to monitor for cases of Bell's palsy following COVID-19 immunization as an increasing percentage of the global population receives vaccination.

## 1 Introduction

Bell's palsy is the clinical syndrome of acute-onset peripheral facial nerve weakness. It is the most common form of facial paralysis, estimated to occur in the general population at a rate of approximately 15–25 per 100,000 person-years.^2^, ^3^, ^4^, ^5^ The precise pathophysiology of Bell's palsy remains unclear but known risk factors include diabetes mellitus and pregnancy.^2^^,^^5^ Cases of Bell's palsy have been reported as adverse events following multiple different vaccinations in scientific literature. Mutsch et al. demonstrated increased risk of Bell's palsy in recipients of an intranasal inactivated influenza vaccine administered in Switzerland during 2000–2001.^6^ The vast majority of research studying the association between other vaccines and Bell's palsy on a large scale has been inconclusive or unsupportive, yet cases have been reported following other inactivated influenza vaccines, Hepatitis A vaccines, meningococcal conjugate vaccines, and others.^3^^,^^7^, ^8^, ^9^ Cases of Bell's palsy following COVID-19 vaccination were reported disproportionately in the vaccine group during phase 3 clinical trials of Pfizer/BioNTech BNT162b2 and Moderna mRNA-1273, and have now been reported multiple times post-licensure in the United States and worldwide.^3^^,^^10^, ^11^, ^12^, ^13^ The U.S. Food and Drug Administration has stated the frequency of Bell's palsy in the vaccine group is consistent with the expected background rate of Bell's palsy in the population, but nevertheless recommends “surveillance for cases of Bell's palsy with deployment of the vaccine into larger populations."^1^ Here we present a case of Bell's palsy following COVID-19 vaccination in an HIV-positive patient, an entity which has not yet been reported. We discuss the diagnostic certainty of this case, potential association to Pfizer/BioNTech BNT162b2 vaccine, and implications of this case in the context of existing research regarding Bell's palsy as an adverse event following immunization.

## 2 Case report

**Fig. 1:**
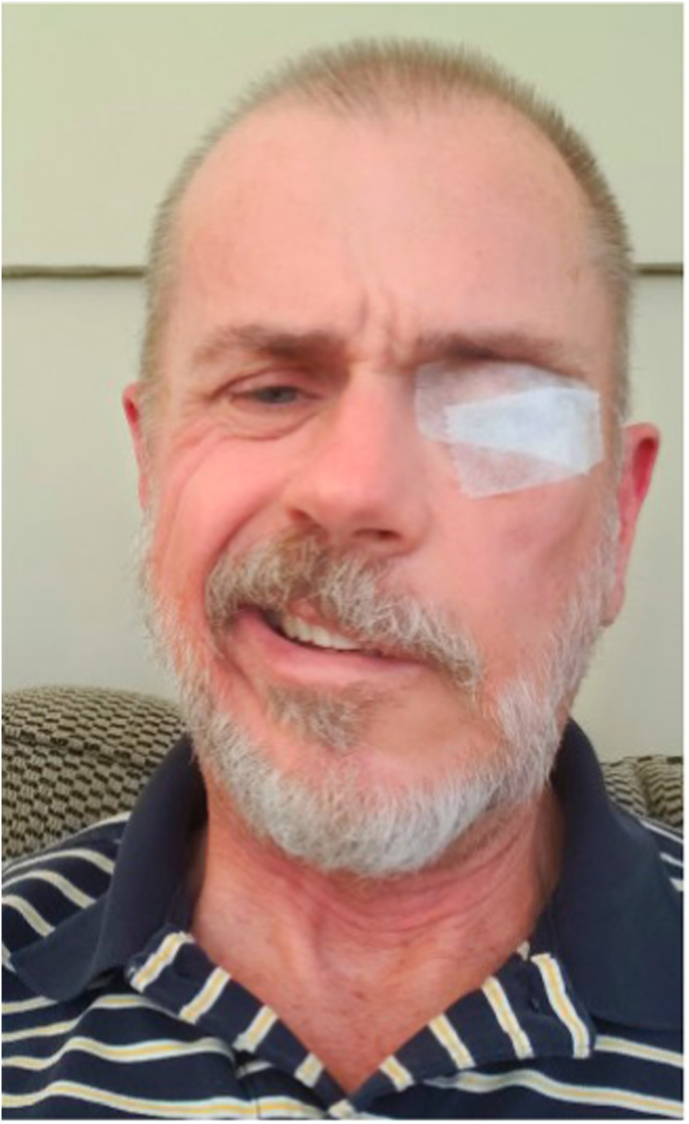
Patient appearance on the day of symptom onset, demonstrating left peripheral facial nerve weakness with forehead involvement.

A 60-year-old male presented to the emergency department for evaluation of left facial droop. He reported spilling water from his mouth during dinner the night before and woke up with persistent left facial weakness and inability to close his left eye. He denied any vision or hearing changes, headache, palpitations, gait disturbance, facial pain, or any other muscular weakness. His past medical history was significant for HIV diagnosed twenty years prior with long-term compliant use of highly active antiretroviral therapy Genvoya (elvitegravir/cobicistat/emtricitabine/tenofovir alafenamide), stage 3 chronic kidney disease, and pre-diabetes. He had received the first dose of Pfizer/BioNTech BNT162b2 vaccination approximately 42 hours prior to symptom onset. Physical examination in the ED revealed left-sided facial weakness with involvement of the forehead, inability to raise left eyebrow, and inability to close left eye with sensation and strength intact in bilateral upper and lower extremities. There were no gait abnormalities, no vesicular skin lesions or other focal neurologic deficits (Fig. 1). Electrocardiogram and laboratory evaluation were within normal limits outside or consistent with baseline laboratory testing. His clinical presentation was judged to be consistent with uncomplicated left-sided Bell's palsy. The patient was discharged with prednisone 60 mg daily for six days and valacyclovir 1000 mg three times daily for 7 days.

**Fig. 2:**
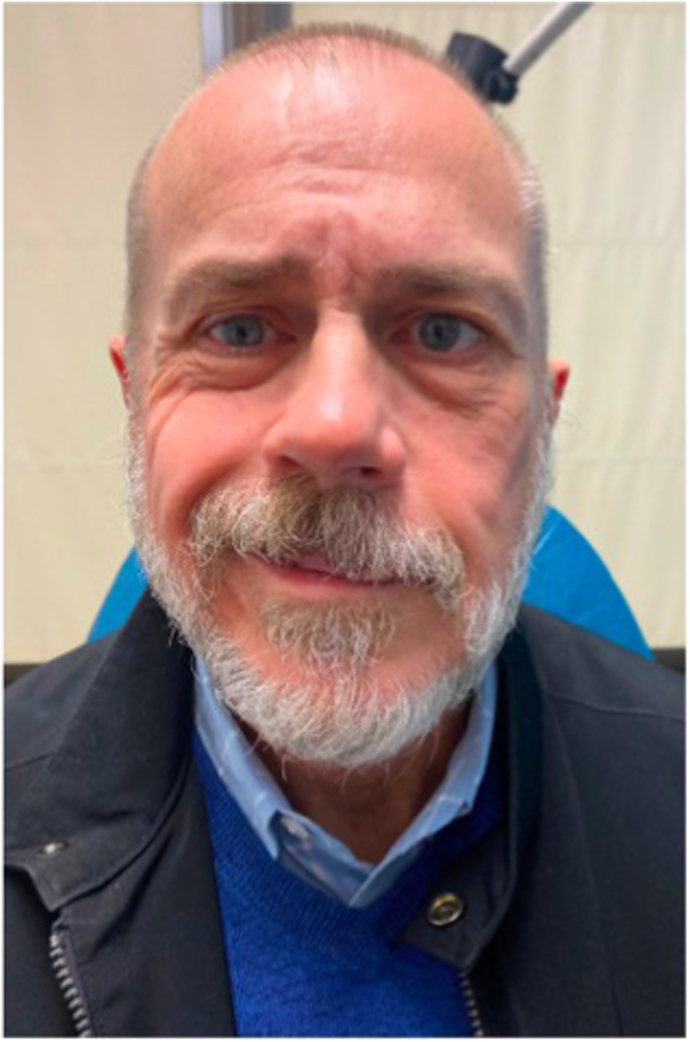
Patient appearance several days after symptom onset, demonstrating improving facial weakness with some return of nasolabial folds and mandibular creases.

**Fig. 3:**
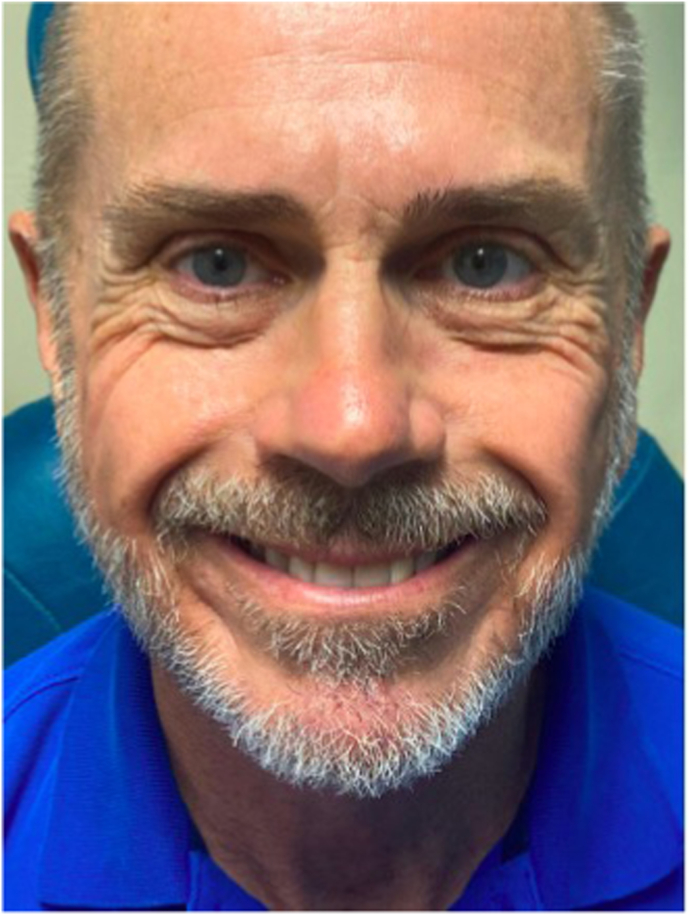
Patient appearance approximately 90 days after symptom onset, demonstrating complete resolution of facial nerve weakness.

The patient was evaluated in our outpatient ophthalmology clinic on day 2 following symptom onset. He complained of left eye irritation and burning sensation, with inability to fully close his left eye. Physical exam was revealing for mild exposure keratopathy, 5 mm lagophthalmos and very poor Bell's reflex in the left eye with otherwise normal exam findings. He was provided ophthalmic lubricating ointment to use hourly, artificial tears as needed, moisture goggles and suggested to tape eyelids nightly. The patient returned to our clinic for follow up at days 5 and 15 following symptom onset and showed marked improvement in his left-sided keratopathy on this treatment regimen, obviating the need for further intervention (Fig. 2). At approximately day 90 following symptom onset, the patient's symptoms and exposure keratopathy had resolved completely (Fig. 3). He received his second dose of Pfizer/BioNTech COVID-19 vaccination as scheduled three weeks following his first dose with no adverse events noted. The patient was encouraged to report this incidence of presumed Bell's palsy following COVID-19 vaccination to the CDC as part of routine vaccine safety monitoring.

## 3 Discussion

Prior to evaluation of potential association between COVID-19 vaccination and Bell's palsy, the diagnostic certainty of Bell's palsy in our case is important to discuss.^4^^,^^7^ Bell's palsy is a peripheral facial nerve weakness, acute in onset, developing to maximal weakness with involvement of the forehead in a period of 24–72 hours. Prognosis is excellent and the majority of patients regain full function within weeks to months. Autoimmune mechanisms and viral infection/inflammation are leading etiological hypotheses, but Bell's palsy remains idiopathic in nature.^2^^,^^4^^,^^14^ Diagnosis is usually clinical, based on characteristic symptoms in the absence of another identifiable diagnosis.

Our patient's clinical presentation was consistent with the above characteristics of Bell's palsy. HIV infection has been linked to increased risk of facial nerve palsy, but this occurs during acute infection and often is accompanied by additional polyneuropathies which was not the case in our patient.^15^^,^^16^ Given his classic presentation with clinical history, physical examination, and laboratory investigations unrevealing for any alternative diagnosis we put forth his diagnosis of Bell's palsy with a high degree of certainty.

In prior scientific literature, an intranasal inactivated influenza vaccine licensed and administered in Switzerland during 2000–2001 has been linked to causation of Bell's palsy. Bell's palsy occurred most commonly during days 31–60 after vaccine administration, with more than three-quarters of cases occurring within 60 days. The relative risk of Bell's palsy following vaccination was roughly 19 times that of control patients. The vaccine is no longer in use.^6^

Seven cases of Bell's palsy occurred during the phase 3 clinical trials of COVID-19 vaccinations conducted in the United States; 4 following administration of Pfizer/BioNTech BNT162b2 and 3 cases following administration of Moderna mRNA-1273 compared to 0 in each control group.^11^^,^^12^ Multiple case reports of Bell's palsy following COVID-19 vaccination have since been published, leading to larger population-based studies.^3^^,^^10^^,^^13^ Current research has produced conflicting results; some studies have demonstrated an increased incidence or risk of Bell's palsy following various COVID-19 vaccinations^17^^,^^18^ while others have not found any association between COVID-19 vaccination and incidence of Bell's palsy.^19^, ^20^, ^21^ Additional large-scale research studies will be necessary to determine the potential association between COVID-19 vaccination and Bell's palsy.

Per FDA recommendations and existing evidence, it will undoubtedly remain important to monitor for development of Bell's palsy symptoms during the 60 days following COVID-19 vaccination.^1^ However, we would like to emphasize that Bell's palsy is a typically benign condition with excellent prognosis. The potential risks of Bell's palsy following vaccination do not currently outweigh the benefits of COVID-19 vaccination.

## 4 Conclusions

Though there is insufficient evidence at this time to support a causal association between COVID-19 vaccines and Bell's palsy, the temporal relationship between vaccination and classic clinical features of Bell's palsy in our patient certainly raises suspicion for association with Pfizer/BioNTech BNT162b2 COVID-19 vaccination. We present this case of Bell's palsy in an HIV-positive patient as an adverse event potentially associated with BNT162b2 COVID-19 vaccination. It will be important to monitor for cases of Bell's palsy following COVID-19 immunization as an increasing percentage of the global population receives vaccination.

## Patient consent

The patient provided written consent for publication of this case.

## Funding

No funding or grant support.

## Authorship

All authors attest that they meet the current ICMJE criteria for authorship.

## Declaration of competing interest

The authors have no conflicts of interest to disclose.
